# Metabolomics Analysis Coupled With UPLC/MS on Therapeutic Effect of Jigucao Capsule Against Dampness-Heat Jaundice Syndrome

**DOI:** 10.3389/fphar.2022.822193

**Published:** 2022-01-28

**Authors:** Yanmei He, Mengli Zhang, Taiping Li, Zhien Tan, Aihua Zhang, Min Ou, Danna Huang, Fangfang Wu, Xijun Wang

**Affiliations:** ^1^ National Engineering Laboratory for the Development of Southwestern EndangeredMedicinal Materials, Guangxi Botanical Garden of Medicinal Plants, Nanning, China; ^2^ National Chinmedomics Research Center, National TCM Key Laboratory of Serum Pharmacochemistry, Chinmedomics Research Center of State Administration of TCM, Laboratory of Metabolomics, Department of Pharmaceutical Analysis, Heilongjiang University of Chinese Medicine, Harbin, China

**Keywords:** metabolomics, ultra-high performance liquid chromatography/mass spectrometry, dampness-heat jaundice syndrome, metabolite, jigucao capsule

## Abstract

Dampness-heat Jaundice Syndrome (DHJS) is a complex Chinese medicine syndrome, while Jigucao capsule (JGCC) is an effective compound preparation of Chinese medicine for the treatment of DHJS about liver and gallbladder, but its mechanism is not clear yet. The purpose of this study is to clarify the pathogenesis of DHJS and the treatment mechanism of JGCC. We used ultra-high performance liquid chromatography/mass spectrometry (UPLC/MS) combined with pattern recognition, accompanied the advanced software and online database for the urine metabolomics of rats. The potential biomarkers disturbing metabolism were identified and the metabolic pathway was analyzed. We investigated the callback of biomarkers after treatment with JGCC. Finally, A total of 25 potential urine biomarkers were identified, including Arachidonic acid, Phenylpyruvic acid, *L*-Urobilin and so on, and 14 related metabolic pathways were disturbed. After treatment with JGCC, the clinical biochemical indexes and histopathological were significantly improved, and the disturbed biomarkers were also obviously adjusted. It is proved that JGCC has remarkable effect on the treatment of DHJS.

## Introduction

Traditional Chinese medicine (TCM) has a long history in the treatment and prevention of diseases (Sun et al., 2018a). Since the outbreak of COVID-19, it has shown the superiority in treating diseases, gaining the international attention to TCM more and more. However, the fuzziness of Chinese medicine syndrome and the complexity of prescriptions have greatly restricted the pace of TCM realizing international modernization (Zhang et al., 2019a). With the rise of metabolomics and its application in the field of TCM, the interpretation for the effective mechanism of Chinese medicine and the precise diagnosis and accurate treatment of clinical syndromes have been effectively solved, and a set of exclusive research methods belonging to TCM have been formed-Chinmedomics. Our team used metabolomics to characterize the metabolic profile and related biomarkers of the heart-qi deficiency syndrome, and revealed the effectiveness of Wenxin formula in treating the heart-qi deficiency syndrome and the material basis of the pharmacodynamics (Wang et al., 2013); basing on the metabolomics technology evaluated the overall effect of Kaixin San in preventing and treating Alzheimer’s disease and identified related biomarkers and metabolic pathways (Chu et al., 2016); using metabolomics to develop Yinchenhao Decoction for the treatment effectiveness of DHJS, and then discover and determine the effective material basis and potential effect targets (Sun et al., 2019a). After more than 10 years of development, the metabolomics research of TCM has achieved remarkable results. Characterizing the overall effect evaluation of TCM/prescriptions from the metabolic profile of diseases/syndromes may change TCM from empirical treatment to modernization with standard and scientific methods (Sun et al., 2014; Qiu et al., 2017; Liu et al., 2018).

DHJS is a common disease in the classification of jaundice from the perspective of TCM theory, also known as Yang Huang syndrome. It was first seen in treatise on febrile diseases, which is one of the jaundice syndromes of hepatobiliary diseases. The manifestations are fever and thirst, the body and eyes are bright yellow as orange, the color of urine is as dark as strong tea, with loss of appetite, nausea and vomiting, difficult defecation, abdominal distension and pain, red tongue and greasy fur and so on (Chen et al., 2014). JGCC is composed of eight botanical drugs such as Abrus melanospermus subsp. melanospermus [Fabaceae; *Abri herba*], Artemisia capillaris Thunb [Asteraceae; *Artemisiae scopariae herba*], Gardenia jasminoides J. Ellis [Rubiaceae; *Gardeniae fructus*], Panax notoginseng (Burkill) F.H.Chen [Araliaceae; *Notoginseng radix et rhizoma*], Paeonia lactiflora Pall [Paeoniaceae; *Paeoniae radix alba*], Origanum vulgare L. [Lamiaceae; *Herba origani*], Ziziphus jujuba Mill [Rhamnaceae; *Jujubae fructus*], Lycium barbarum L [Solanaceae; *Lycii fructus*] and two animal materials. It has the effects of soothing the liver and gallbladder, clearing away heat and detoxification. JGCC is a good medicine for the treatment of hepatitis and cholecystitis (Zhao et al., 2009) and contains a large number of chemical components such as alkaloids, flavonoids, terpenes and organic acids. In the early stage, we have systematically studied the chemical components of JGCC, and 144 compounds have been identified (Li et al., 2021a). JGCC used in this study is a commercial drug. Before leaving the factory, strict quality control is carried out according to the enterprise standard, including character description, thin layer chromatography identification with Artemisia capillaris Thunb [Asteraceae; *Artemisiae scopariae herba*] is used as the control drug and the moisture content shall not exceed 7%. The quality control is effectively guaranteed, but the quantitative control of chemical components is not carried out. Corresponding research should be carried out to make up for deficiencies. We applied the advanced UPLC-G2Si-HDMS instrument system, combined with the metabolomics strategy, to explore the disturbed metabolic markers and pathways, determine the pathogenesis of DHJS (Sun et al., 2018b). We also analyzed the therapeutic effect of JGCC on DHJS from the perspective of metabolites and clarified the treatment mechanism. The results provided a basis for the establishment of quantitative standard of JGCC.

## Materials and Methods

### Instrument

Acquity ultra high performance liquid chromatograph (Waters, United States); Synapt ™ G2Si High definition mass spectrometer (HDMS) (Waters, United States); Sorvall ST-8R high speed freezing centrifuge (Thermo Fisher, United States); GL6231-1SCN electronic analytical balance (Sartorius Scientific instrument Co., Ltd.); Hitachi 3100 automatic biochemical analyzer (Nanning Precision Instrument Co., Ltd.); Tecan infinite M200 pro multifunctional microplate reader (Switzerland); DHG-9140A electric constant temperature blast drying oven (Shanghai Qixin Scientific Instrument Co., Ltd.); SB-800DT ultrasonic cleaning machine (Ningbo Xinzhi Biotechnology Co., Ltd.); Vortex-6 vortex instrument (Macao slinberg Instrument Manufacturing Co., Ltd.); RVC2-18CD plus vacuum concentrator (Beijing gaodetong Technology Co., Ltd.).

### Drugs and Reagents

Chromatographic grade methanol, acetonitrile and formic acid (Thermo Fisher company); Distilled water (Guangzhou Watsons Food and Beverage Co., Ltd.); α-Naphthalene isothiocyanate (ANIT) (Shanghai Chaoyan Biotechnology Co., Ltd.); ethanol (Sinopharm Chemical Reagent Co., Ltd.); Zingiber officinale Roscoe [Zingiberaceae; *Zingiberis rhizoma*] (Guangxi Xianzhu TCM Technology Co., Ltd.) was authenticated by Prof. Xi-jun Wang of the Pharmacognosy Department, Heilongjiang University of Chinese Medicine; Normal saline (Guangxi Yuyuan Pharmaceutical Co., Ltd.); JGCC (Guangxi Yulin Pharmaceutical Group Co., Ltd.); Olive oil (Beijing Hualian Supermarket); Paraformaldehyde (Beijing Labgic Technology Co., Ltd.); Pentobarbital sodium (Beijing boatuoda Co., Ltd.); Sodium formate (Sigma, United States); Leucine enkephalin (waters, United States). Assay kit for Alanine aminotransferase (ALT), Aspartate aminotransferase (AST), Alkaline phosphatase (ALP), Total bile acid (TBA), Superoxide dismutase (SOD), Prealbumin (PA) purchased from Zhongsheng Beikong Biotechnology Co., Ltd., the kit for Total bilirubin (T-Bili) and Direct bilirubin (D-Bili) obtained from Shanghai Lanpai Biotechnology Co., Ltd.

### Experimental Animal

SD rats (SPF grade), male, weighing 180 ± 20 g; feeding environment (temperature: 24 ± 2°C, humidity: 65–75%), purchased from Guangxi Medical University. 12 h are the cycle of alternating light and dark, drinking and eating freely, and the experiment begins after a week of adaptation. Animal license number is SYXK (Gui) 2020−0014.

### Preparation of Modeling Solution

300 g of Zingiber officinale Roscoe [Zingiberaceae; *Zingiberis rhizoma*] was weighed, soaked in 3,000 ml distilled water for 1 h, heated to boil and then with slow fire for 1 h, filtered and added 3,000 ml distilled water to the drug residue, the above operation was repeated twice, the filtrate of three times was collected and concentrated to 3,000 ml, and the 0.013 g/ml Zingiber officinale Roscoe [Zingiberaceae; *Zingiberis rhizoma*] modeling solution was prepared by taking appropriate amount of concentrated solution and adding water. An appropriate amount of absolute ethanol was taken, distilled water was added and shaken well to prepare a 12.5% (V/V) ethanol modeling solution. Weighed 130.0 and 90.0 mg of ANIT powder, added 50 ml of olive oil, mixed well, and dissolved by ultrasound to prepare ANIT modeling solutions with concentrations of 2.6 and 1.8 mg/ml.

### Animal Grouping and Administration

24 preconditioned adult male SD rats were randomly divided into three groups with eight rats in each group: blank control group, model control group and JGCC group. The rats of the model and JGCC group were given Zingiber officinale Roscoe [Zingiberaceae; *Zingiberis rhizoma*] solution according to the dose of 0.7 ml/200 g in the morning and 12.5% ethanol solution in the afternoon according to the dose of 1.0 ml/100 g for 14 days; ANIT olive oil solution was given at the doses of 10.4 and 7 mg/kg on the 15th and 16th days respectively, and the blank control group was given the same volume of distilled water and olive oil. From the 17th day, the JGCC group was given JGCC solution (dissolved in distilled water) at the dose of 2.16 g/kg, while the blank control group and model control group were given equal volume of distilled water, successive administration 14 days.

### Collection and Processing of Biological Samples

One hour after the last administration in the morning of the 30th day, the rats were anesthetized with 2% pentobarbital sodium. Blood samples were collected from the abdominal aorta, and the fresh blood was centrifuged after 30 min (4°C, 4,000 rpm, 15 min), the supernatant was stored in the refrigerator at −80°C. After collecting the blood, the rat liver was immediately removed, the residual blood was washed with normal saline, and fixed in 4% paraformaldehyde solution for histopathological observation. Meanwhile, the urine of rats at night for 12 h was collected on days 0, 14, 16, 23, 30. The fresh urine was centrifuged (4°C, 4,000 rpm, 15 min) and the supernatant was stored in the refrigerator at −80°C.

### Data Acquisition

#### Chromatographic Condition

The mobile phase is gradient eluted with 0.1% formic acid water (A) and 0.1% formic acid acetonitrile solution. The elution procedures is 0–3 min, 99-90%A; 3–5min, 90-80%A; 5–8.5 min, 80-60%A; 8.5–9.5 min, 60-1%A; 9.5–11.5 min, 1-1%A; 11.5–12 min, 1-99%A; 12–14 min, 99-99%A. Chromatographic column: Waters ACQUITY UPLC^®^ HSS T3 (2.1 mm × 100 mm, 1.8 μm); Column temperature: 40°C; Sample room temperature: 10°C; Flow rate: 0.4 ml/min; Injection volume: 2 μl; The effluent from the chromatograph is directly injected into the mass spectrometer without shunt for positive and negative ion scanning analysis.

#### Mass Spectrum Condition

Electronic spray ionization (ESI) source; Capillary voltage 2,000 V; Taper hole voltage 40 V; Ion source temperature 105°C; Desolvation gas temperature 400°C; Taper hole gas flow rate 50 L/h; Desolvation gas flow 800 L/h; High collision energy 20–30 V; Low collision energy 6 V. The accurate mass determination was determined by using the solvent of leucine-enkephalin ([M + H]^+^ = 556.2771 [M-H]^-^ = 554.2615) as the locking mass solution. Full scanning in MS^E^ mode is adopted, and the mass scanning range is m/z = 50–1,500 Da.

#### Preparation of Sample Solution

The rat urine sample was thawed at room temperature. The original concentration of rat urine was mixed with distilled water at 1:4, vortex for 10 s, centrifuged (4°C, 13,000 rpm for 10 min) and the supernatant was taken for UPLC-G2Si-HDMS analysis. At the same time, 10 ul of urine collected from the blank group, model group and JGCC group on days 0, 14, 16, 23, and 30 respectively were mixed and shaken to get the quality control samples for reference in the whole collection process.

### Data Statistics and Analysis

Import all original data into Progenesis QI software (Waters). After the software automatically identifies peaks and reduces dimensionality, it takes the voluntarily selected optimal sample data as a reference, and the other samples are matched with it for peak alignment, the matching degree must be greater than 80%, and then carry out experimental grouping and set the extraction conditions such as retention time for peak extraction and deconvolution. At the same time, the Human Metabolome database (HMDB) was selected for compound identification. Finally, the matrix containing retention time, M/Z and normalized peak area was output. After the above data processing of peak picking, dimensionality reduction, peak extraction and identification, unsupervised principal component analysis (PCA) is performed on each group of data by using nested ezinfo 3.0 module, score plot reflecting the clustering degree of each group was obtained. The urine metabolic profile data of model group and blank control group were analyzed by orthogonal partial least squares discriminant analysis (OPLS-DA), the VIP plot reflecting the contribution of variables was obtained, and the variable ions with VIP>1.0 and inter group *t*-test *p* < 0.05 were selected as the set of potential biomarkers; Furthermore, the biomarkers of dampness-heat jaundice rats were determined by further structural identification using secondary mass spectrometry information combined with HMDB (https://hmdb.ca/), KEGG and other omics databases. The associated metabolic pathway of endogenous biomarkers was constructed by MetPA database (Xiong et al., 2021). The callback degree of biomarkers in JGCC group was observed, and the biomarkers with significant callback were selected as effective biomarkers to evaluate the therapeutic effect of JGCC on rats with dampness-heat jaundice.

## Result

### Clinical Biochemical Indexes

**FIGURE 1:**
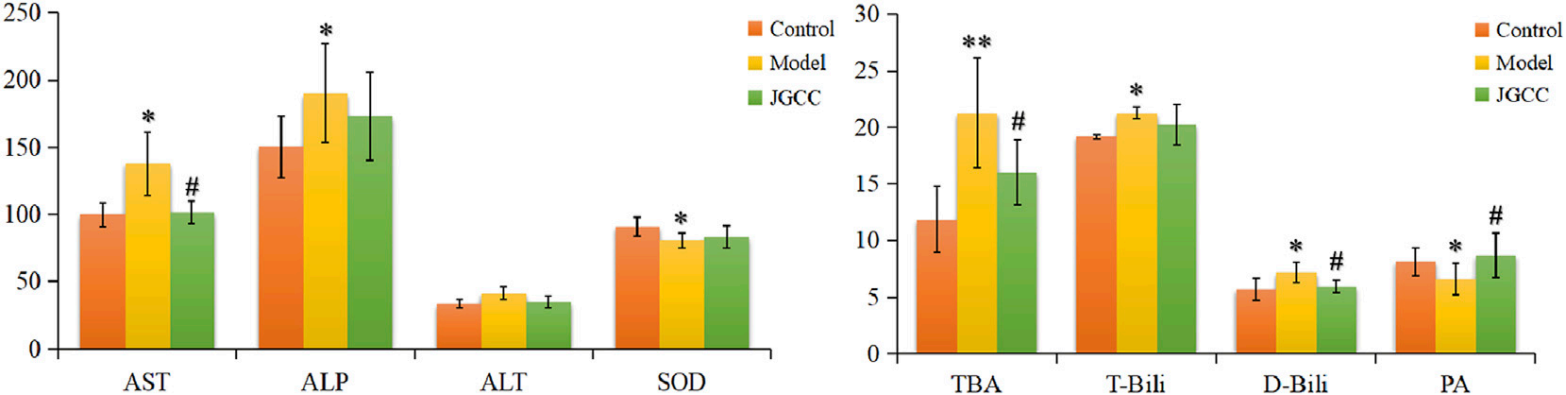
Biochemical indexes analysis of the therapeutic effects of JGCC against DHJS. *p < 0.05, **p < 0.01 vs Control, ^#^ *p* < 0.05, ^##^ *p* < 0.01 vs Model. The corresponding parameters are represented to the supplementary table S1.

According to the instruction of the kit, the content of clinical biochemical indexes in serum of rats in each group were measured, including liver function indexes AST, ALT, PA, liver oxidation index SOD, bile function indexes ALP, TBA, T-Bili, D-Bili. The results are shown in Figure 1 and Supplementary Table S1. Compared with the control group, TBA in the model group increases extremely (*p* < 0.01), AST, ALP, T-Bili and D-Bili all have a significant upward (*p* < 0.05), ALT has an increase trend, SOD and PA decrease prominently (*p* < 0.05). T-Bili and D-Bili are common clinical diagnostic indicators of jaundice, which can directly reflect the occurrence of obstructive jaundice; The above results show that the model group had severe cholestasis and obvious liver injury, indicating that the dampness-heat jaundice model was successfully established (Sun et al., 2019a; Fang et al., 2020). Compared with the model group, JGCC group has a tendency to adjust all the indexes, and there are visible differences in AST, TBA, D-Bili and PA (*p* < 0.05), which proves that JGCC has a good therapeutic effect on liver injury in rats with DHJS.

### Histopathology

**FIGURE 2:**
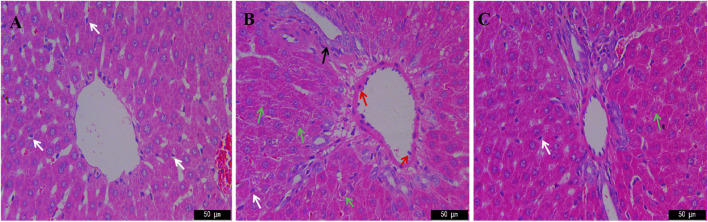
HE staining in the therapeutic effect of JGCC against DHJS with microscope (×400). **(A)** Control; **(B)** Model; **(C)** JGCC. white arrows: kupffer cells; red arrows: inflammatory cells; green arrows: hepatocyte necrosis; black arrows: swelling of vascular endothelial cells.

Liver HE staining results of rats in each group are shown in Figure 2. The liver cells of the blank group are tightly arranged with complete structure. There is no inflammatory cell aggregation around the interlobular veins. Kupffer cells are scattered among hepatocytes and refer to phagocytes located in the hepatic sinuses, which can clear foreign antigens, antigen-antibody complexes and cell debris in the blood, and are the guardians of the human body. Compared with the blank group, the hepatic cells in the model group were disorganized, with edema around the portal area, degeneration and necrosis of hepatic cells, nucleolysis, and decrease of kupffer cells, suggesting that the model of dampness-heat jaundice was successfully prepared (Fang et al., 2020). Compared with the model group, the liver cells of JGCC group are arranged neatly, the degree of necrosis is weaker than that of the model group, the number of kupffer cells is similar to that of the blank group, and the liver injury is significantly improved.

### Metabolic Profile and Biomarkers

**FIGURE 3:**
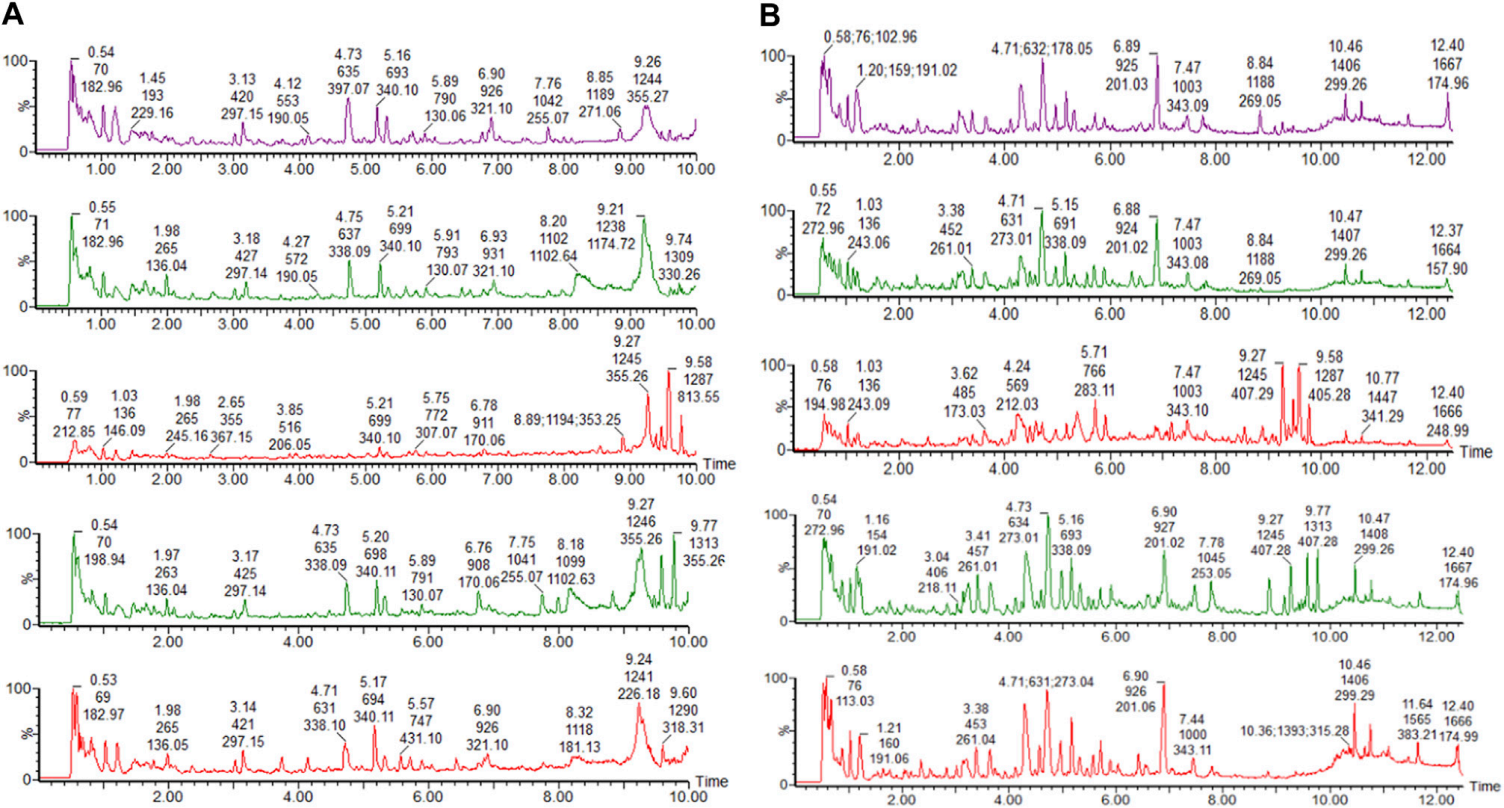
Urine chromatogram of rats in the model group on days 0, 14, 16, 23, 30 (from top to bottom). **(A)** positive ion mode; **(B)** negative ion mode.

**FIGURE 4:**
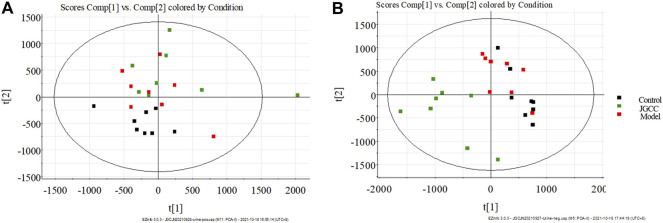
PCA score plots of rat urine samples. **(A)** positive ion mode; **(B)** negative ion mode.

The clinical biochemical indexes and histopathological results show that the rat model of dampness-heat jaundice was successfully established with the combined solution of Zingiber officinale Roscoe [Zingiberaceae; *Zingiberis rhizoma*], ethanol and ANIT. Urine TIC chromatogram showed that the urine metabolic profile of model group rats changed obviously, as is shown in Figure 3. The urine data was collected with UPLC-G2Si-HMDS on the 30th day and analyzed by Progenesis QI software. After standardized treatment, the score plot of PCA analysis is shown in Figure 4. It can be seen from the figure that the blank group and the model group are separated from each other, indicating that the metabolic network in the model group has changed. In the negative ion mode, the JGCC group is significantly far away from the model group, indicating that there is a difference between the JGCC group and the model group; In the positive ion mode, it is seen that the JGCC group is between the blank group and the model group, suggesting that JGCC can delay the pathological process of dampness-heat jaundice rats to a certain extent. Since PCA analysis is an unsupervised mode and belongs to exploratory analysis, and metabolomics data is extremely complex, simple unsupervised analysis could not well distinguish the group differences between samples, we continue to use supervised analysis.

**FIGURE 5:**
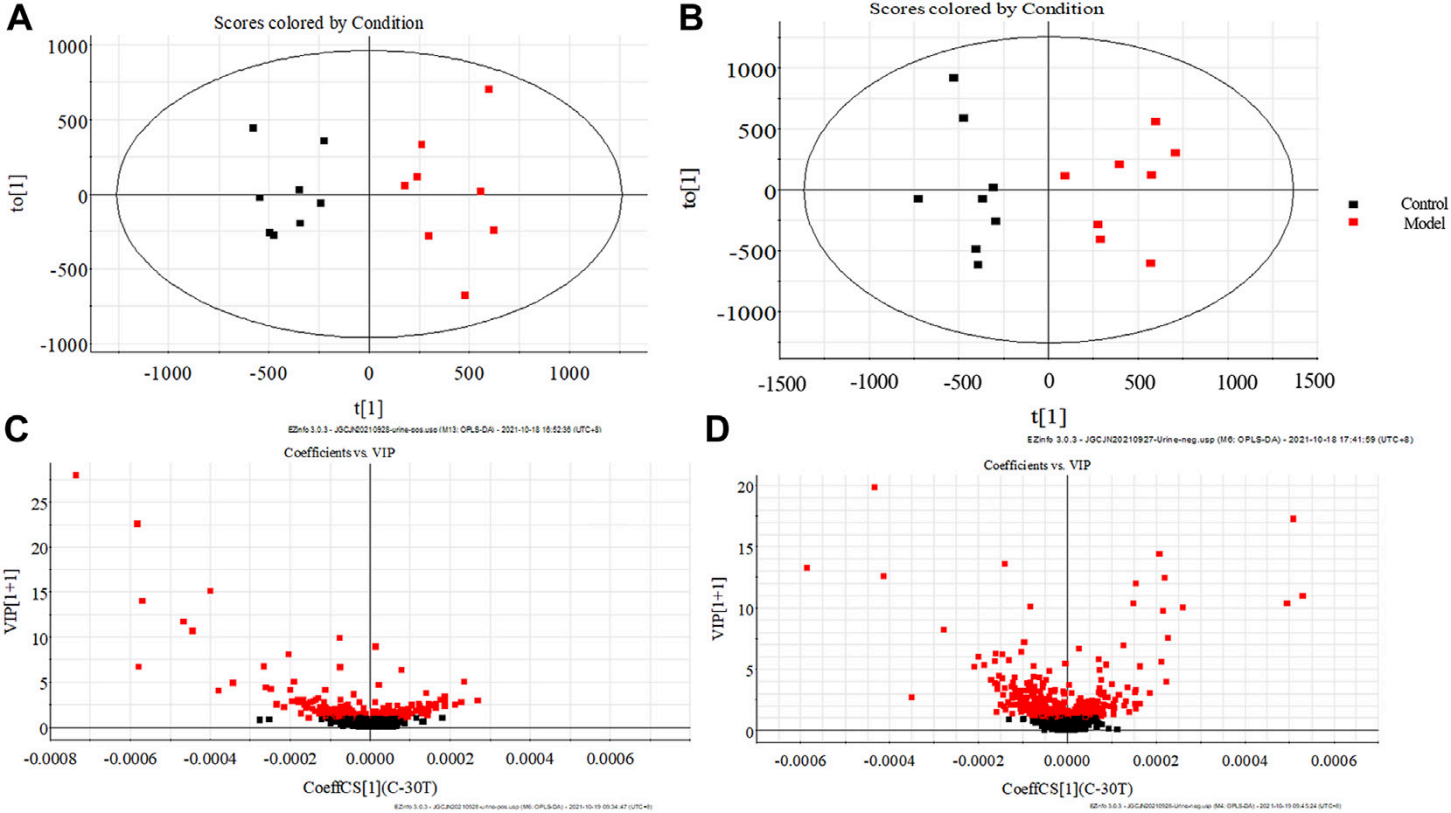
OPLS-DA score plots of rat urine in control group and model group **(A)** positive ion pattern; **(B)** negative ion pattern. VIP scatter plots of rat urine in control group and model group. **(C)** positive ion pattern; **(D)** negative ion pattern.

**FIGURE 6:**
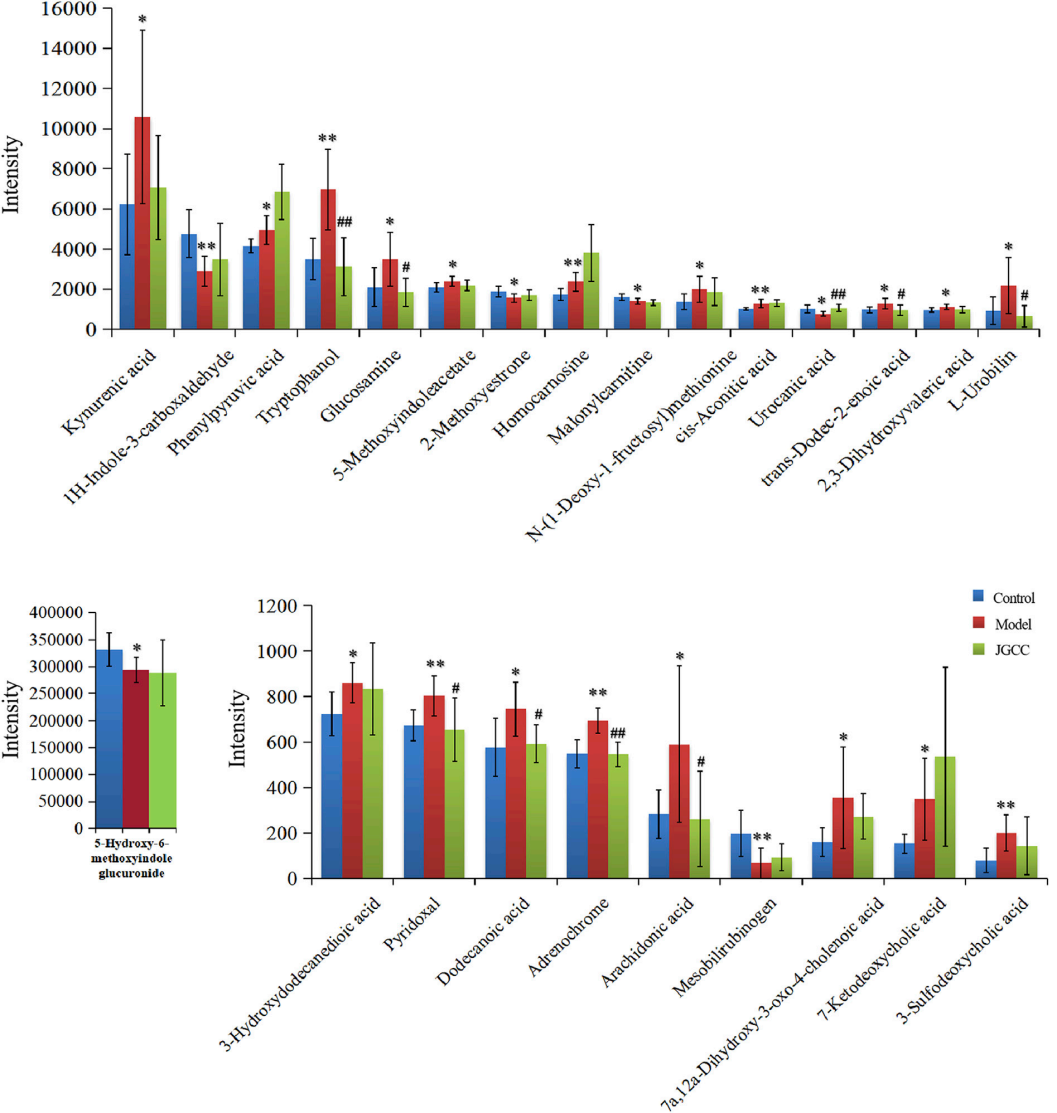
Relative expression intensity of potential biomarkers in control, model and JGCC group. *p < 0.05, **p < 0.01vs Control; ^#^ *p* < 0.05, ^##^ *p* < 0.01vs Model.

The urine data of the model group and the blank group were further analyzed by the supervised OPLS-DA method. The farther away from the origin of the VIP Scatter plot, the greater contribution rate of ions. The variable ions with VIP>1 and *t*-test *p* < 0.05 were selected as potential biomarkers for structural identification, 25 potential biomarkers of dampness-heat jaundice rats were identified, See Figure 5 and Supplementary Table S2. These potential biomarkers disturbed the normal metabolism of dampness-heat jaundice rats and led to metabolic abnormalities. 25 biomarkers were significantly different between the model group and the blank group, while 19 biomarkers in the JGCC group were callback and 9 of them showed significantly, among them, Tryptophanol, Glucosamine, Urocanic acid, *trans*-Dodec-2-enoic acid, *L*-Urobilin, Pyridoxal, Dodecanoic acid, Adrenochrome, and Arachidonic acid could all return to the same level as the blank group. It is indicated that JGCC can prominently inhibit the metabolic disorder of rats with dampness-heat jaundice and has obvious therapeutic effect, as shown in Figure 6 and Supplementary Table S3.

### Metabolic Pathway Analysis

**FIGURE 7:**
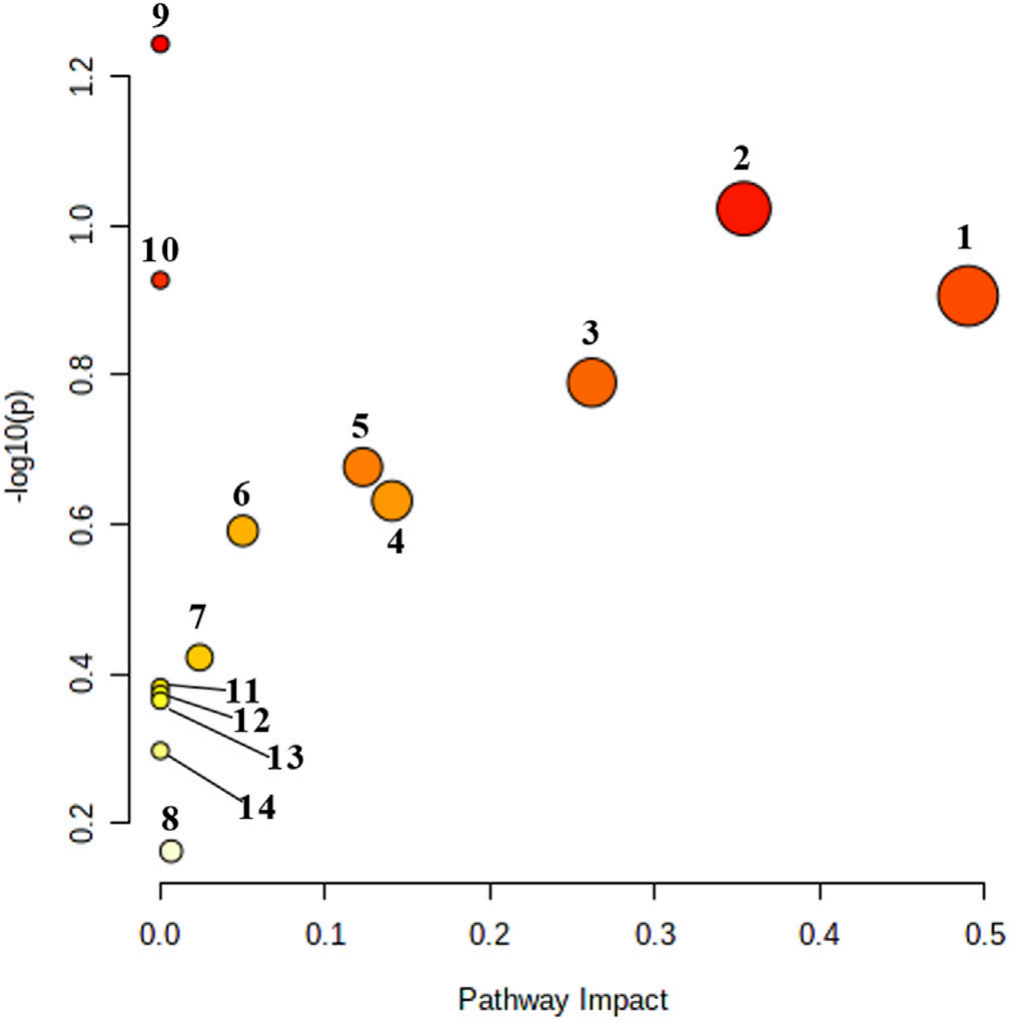
Urine metabolic pathway analysis results. 1: Vitamin B6 metabolism; 2: Arachidonic acid metabolism; 3: Phenylalanine metabolism; 4: Pentose and glucuronate interconversions; 5: Histidine metabolism; 6: Citrate cycle; 7: Glyoxylate and dicarboxylate metabolism; 8: Steroid biosynthesis; 9: Phenylalanine, tyrosine and tryptophan biosynthesis; 10: Tryptophan metabolism. 11: Biosynthesis of unsaturated fatty acids; 12: Amino sugar and nucleotide sugar metabolism; 13: Arginine and proline metabolism; 14: Fatty acid biosynthesis.

**FIGURE 8:**
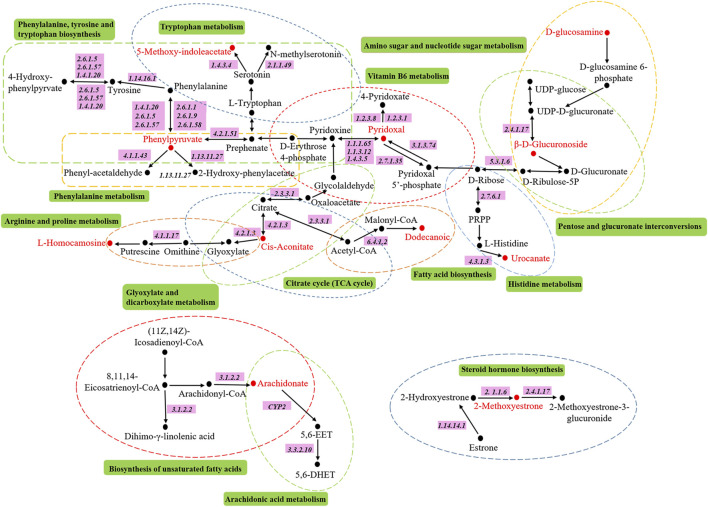
The metabolic pathways associated with all potential biomarkers in KEGG. Red represents the biomarkers detected in our research.

The metabolic pathways related to biomarkers were constructed by using MetPA online database, we constructed 14 metabolic pathways, including phenylalanine, tyrosine and tryptophan biosynthesis, arachidonic acid metabolism, tryptophan metabolism, vitamin B6 metabolism, phenylalanine metabolism, histidine metabolism, pentose and glucuronate interconversions, citrate cycle, glyoxylate and dicarboxylate metabolism, biosynthesis of unsaturated fatty acids, amino sugar and nucleotide sugar metabolism, arginine and proline metabolism, fatty acid biosynthesis and steroid hormone biosynthesis, as shown in Figure 7 and Figure 8. The results show that endogenous metabolites interfere with the metabolic pathways related to DHJS; among them, vitamin B6 metabolism, arachidonic acid metabolism, phenylalanine metabolism, pentose and glucuronate interconversions, histidine metabolism, citrate cycle, glyoxylate and dicarboxylate metabolism, steroid hormone biosynthesis have a greater effect on metabolic disorder in rats with dampness-heat jaundice.

## Discussion

Due to the changes of health mode and disease spectrum, a single traditional clinical biochemical index can no longer meet the diagnosis of complex system diseases in today’s society (Wang et al., 2012), especially TCM syndromes, such as DHJS, is the process of complex pathological changes of the body in the hot and humid environment, mainly involving the liver and other organs. In recent years, the emerging metabolomics technology can use specific biomarkers to represent complex TCM syndromes, and the markers are in an important position of relevant metabolic pathways have an irreplaceable position. UPLC/MS is a mature and effective detection method for compounds. It ionizes samples into gaseous ion mixtures and separates them according to their m/z. Commonly used ion sources are Electron impact ionization (EI), ESI, Atmospheric pressure chemical ionization (APCI) and so on; Which is mainly used for the analysis of macromolecular organic compounds with high polarity, thermal instability and difficult volatilization, or compounds that are difficult to be analyzed by gas chromatography mass spectrometrometry. At present, the liquid chromatography electrospray ionization mass spectrometry system has been widely used in high-throughput drug analysis. It can improve analytical speed, selectivity, sensitivity and reliability. About 80% of the compounds can be detected by mass spectrometry after chromatographic separation; It is also possible to identify the metabolic components in biological samples and detect endogenous metabolic markers associated with disease occurrence in vivo. For example, Postmenopausal osteoporosis (PMOP) is a common clinical disease in postmenopausal women, UPLC/MS method was used to identify serum metabolites, and finally 16 potential biomarkers were identified, which is helpful to clarify the pathological mechanism of PMOP rats (Zhang et al., 2019b); The researchers used ultra-high performance liquid chromatography-quadrupole time of flight-tandem mass spectrometry to screen 33 metabolites related to alcoholic liver injury (ALD) for clarifying the pathogenesis of ALD (Qiu et al., 2020); Coronary heart disease (CHD) is a relatively complex disease, there is no effective method for early diagnosis and prevention, The metabolomics technology based on UPLC-HDMS can clarify the metabolic spectrum, biomarkers and related metabolic pathways of CHD model. The researchers eventually determined 25 biomarkers and 9 related metabolic pathways associated with CHD (Sun et al., 2019b). Therefore, the combination of UPLC/MS and metabolomics technology can comprehensively understand TCM syndromes from the perspective of multi-level and multi-target, and then thoroughly understand the mechanism of TCM in the treatment of diseases (Fang et al., 2016).

Ethanol was used to produce wet background, Zingiber officinale Roscoe [Zingiberaceae; *Zingiberis rhizoma*] was used to produce heat background, and combined with ANIT was used to prepare the rat model of dampness-heat jaundice in this study, which was evaluated by classical diagnostic indexes. At the same time, the histopathological examination of the liver preliminarily showed that the rats with dampness-heat jaundice were successfully prepared, and the liver injury was significantly improved after treatment with JGCC. This modeling method has been very mature, adopted by many scholars and recognized by the majority of researchers (Fang et al., 2016; Fang et al., 2020; Liu et al., 2018; Sun et al., 2019a; Xiong et al., 2021). The therapeutic dose of JGCC was calculated according to the daily dose of 85.71 mg/kg for human. In the pretreatment experiment, we carried out the rat pharmacodynamic test of low, medium and high doses of JGCC. The doses were 0.54, 1.08, and 2.16 g/kg respectively, which were calculated according to 1, 2 and 4 times of the equivalent dose of human administration; Through the improvement of biochemical indexes and liver histopathology, it was found that the therapeutic effect of high-dose group was better than that of low and medium dose groups. Therefore, the dosage of the treatment group we chose 2.16 g/kg. On this basis, advanced UPLC-G2Si-HDMS technology combined with pattern recognition method was used to identify the phenotype of DHJS, and 25 key urine metabolites were identified as potential biomarkers, which play an important role in the regulation of metabolic network.

Studies have shown that one of the important pathways involved in severe liver injury in the pathogenesis of DHJS is the pentose and glucuronate interconversions (Zhang et al., 2016a; Fang et al., 2016; Sun et al., 2018b; Liu et al., 2018; Sun et al., 2019a). In the liver, glucuronic acid combines with lipophilic poisons through glycosidic bonds to form hydrophilic substances that are excreted from the body in the bile or kidney. This process can be used to evaluate the detoxification ability of the liver. Uridine diphosphate glucose produced during glucose metabolism is further oxidized to uridine diphosphate glucuronate (UDPGA), under the catalysis of uridine diphosphate glucuronosyltransferase 1A1 (UGT1A1, EC2.4.1.17), UDPGA can combine glucose with hydroxyl or amino of toxic substances, so as to increase water solubility and facilitate the excretion of toxic substances. Bilirubin is produced by red blood cells. When red blood cells age, lipophilic indirect bilirubin will be released and enter liver tissue with blood circulation and be converted into direct bilirubin with high hydro-soluble under the action of glucuronic acid, which is excreted from the body (Roche and Kobos., 2004). UGT1A1 is an important metabolic enzyme, which can not only catalyze the glucuronidation of drugs, poisons, steroids and thyroid hormones, promote the biosynthesis of glucosides in the brain, but also participate in the synthesis of endogenous compounds such as bilirubin, bile acids and short chain fatty acids (Rowland et al., 2013). Through focusing analysis, it is found that UGT1A1 is the only bilirubin metabolic enzyme located the upstream of d-glucuronic acid in the metabolic pathway of pentose and glucuronate interconversions. Indirect bilirubin must be transformed into direct bilirubin through UGT1A1 (Memon et al., 2016). It combines with Y protein and Z protein to produce highly water-soluble bilirubin-Y protein and bilirubin-Z protein, which are dissolved in bile and then excreted from the body. Therefore, any error of pentose and glucuronate interconversions will lead to an increase in the assay of bilirubin in serum, and the obvious clinical manifestations is that skin and mucosa are as yellow as orange (Fang et al., 2020). It is found that 5-Hydroxy-6-methoxyindole glucuronide is a product of UDPGA under the action of UGT1A1. In our study, we found that the 5-Hydroxy-6-methoxyindole glucuronide content in the urine of model rats was significantly lower than blank group, which proves that the metabolism of model rats is abnormal, resulting in the disorder of the pentose and glucuronate interconversions, further leading to the occurrence of dampness-heat jaundice.

Arachidonic acid is a ω-6 polyunsaturated fatty acids mainly exists on the cell membrane in the form of phospholipids and plays an important biological role in the liver (Li et al., 2021b; Zhao et al., 2021). The metabolites of arachidonic acid, as lipid mediators, play an important role in regulating the physiological function and pathology of the liver, such as prostaglandin E2, prostacyclin, thromboxane A2 and leukotriene C4. When liver injury occurs, macrophages gather in the liver and activate, resulting in the release of a large number of arachidonic acid metabolites, such as toxic prostaglandins, leukotrienes, TXs, free radicals and tumor necrosis factor-α, these toxic substances directly lead to the damage of hepatocytes (Chen et al., 2011; Sun et al., 2018a; Sztolsztener et al., 2020). Arachidonic acid, as an important inflammatory lipid mediator, regulates oxidative stress in hepatocyte mitochondria through three metabolic pathways of cyclooxygenase, lipoxygenase and cytochrome P450, resulting in the oxidation of a large number of fatty acids and the formation of lipid peroxides, the deposition of collagen in the liver, aggravating the injury of hepatocytes and the activation of hepatic stellate cells, and finally accelerating the injury of hepatocytes (Sacerdoti et al., 2003; Tallima, 2021). In our study, the content of arachidonic acid in the urine of model group was dramatically higher than blank control group. It proves that the metabolism of arachidonic acid is abnormal, and its metabolic pathway is disordered; a large number of inflammatory cell infiltration can be found in the pathological tissue section, which further supports the abnormal metabolism of arachidonic acid. After the treatment of JGCC, the content of arachidonic acid in rat urine was significantly reduced and returned to the blank group, proving that the JGCC has a obvious therapeutic effect.

The liver is the main metabolic site of phenylalanine. Under normal circumstances, phenylalanine in the body is mainly metabolized to tyrosine with the catalysis of enzymes (Tessari et al., 2010). When the liver has pathological changes, phenylalanine metabolism is disordered, resulting in abnormal metabolism, which leads to generate phenylpyruvic acid and phenylacetic acid by transamination with the blood circulation entering the systemic circulation, the clinical manifestation is called phenyl ketonuria (Liu et al., 2018; Fang et al., 2016). Due to the increase of phenylalanine and ketones in the blood, some hepatocytes are damaged and substances in hepatocytes are released into the blood, including aspartate aminotransferase (AST). AST is an essential substance for the metabolic transformation of phenylalanine and phenylpyruvic acid, and it is also one of the gold indexes reflecting liver function in clinic. Phenylpyruvic acid is a direct metabolite of phenylalanine, and its amount is closely related to tyrosinemia. The increase of phenylpyruvic acid content in urine reflects the abnormal metabolism of phenylalanine, suggesting that intracellular tyrosine aminotransferase causes hepatocyte injury and tyrosinemia (Zhang et al., 2016b; Sanaei Dashti and Sedigheh Hamzavi, 2019). Therefore, tyrosinemia and abnormal metabolism of AST can promote the occurrence of jaundice syndrome. In this study, the content of phenylpyruvic acid in urine of rats with dampness-heat jaundice was significantly higher than blank group, suggesting that phenylalanine metabolism is abnormal and its metabolic pathway is disturbed. This result is proved by the significant increase of clinical biochemical index AST. The content of AST decreased prominently and was pulled back to the level of blank group with the treatment of JGCC.

Vitamin B6 is a nitrogen-containing compound, mainly in three natural forms: pyridoxal, pyridoxol, pyridoxamine and their phosphoric acid derivatives such as Pyridoxal-5-phosphate (PLP), pyridoxol-5-phosphate (PNP), pyridoxamine -5-phosphate (PMP), which are abundant in the liver. The liver is an active tissue for vitamin B6 metabolism. Vitamin B6, a component of some coenzymes in human body, is involved in various metabolic reactions, especially the metabolism of amino acids. It is closely related to nearly hundred enzyme reactions, including the metabolism of all amino acids, such as heme metabolism and tryptophan metabolism, as well as the biosynthesis of some neuromediums and DNA (Mitchell et al., 1976; Bitetto et al., 2011). Relevant studies have shown that AST requires PLP as a coenzyme to express its activity. Patients with liver disease have abnormal PLP regulation. Therefore, aberrant metabolism of vitamin B6 will directly lead to disordered operation of relevant metabolic network in the body, and ultimately result in the occurrence of jaundice. In our study, the content of pyridoxal in the urine of rats with dampness-heat jaundice was significantly higher than that of the blank group, which proved that the metabolism of vitamin B6 in the model group was abnormal and caused the appearance of dampness-heat jaundice. After treatment with JGCC, the content of pyridoxal in urine returned to the normal level.

## Conclusion

In this study, we established a rat model of DHJS according to the basic theory of TCM. The success of model preparation was preliminarily proved by classical clinical biochemical indexes and liver histopathology. On this basis, 25 urine biomarkers of rats with dampness-heat jaundice were successfully identified by advanced UPLC-G2Si-HDMS technology combined with metabolomics. These markers are considered as possible drug targets, and 14 related metabolic pathways are determined. After treatment with JGCC, the disordered metabolic pathways are regulated successfully. Meanwhile, we have carried out a detailed biological elaboration on several major pathways with high impact value in metabolic pathways, and combined with the analysis of related metabolites in urine, the pathogenesis of dampness-heat jaundice and the therapeutic mechanism of JGCC have been clarified. It is proved that urine metabolomics is a favorable tool for in-depth study of TCM syndromes, and provides a promising strategy for JGCC in the treatment of DHJS.

## Data Availability

The original contributions presented in the study are included in the article/Supplementary Material, further inquiries can be directed to the corresponding authors.
