# Supplementary material for: Metabolomics Analysis Coupled With UPLC/MS on Therapeutic Effect of Jigucao Capsule Against Dampness-Heat Jaundice Syndrome
Source: Front Pharmacol. 2022 Jan 28;13:822193. doi: 10.3389/fphar.2022.822193 (PMC8831696; doi:10.3389/fphar.2022.822193)
Supplement: Supplementary file 1 [file Table1.docx]

**Table S1** Analysis results of clinical biochemical indexes (mean ±SD, n= 8)

| **Group**  **Index** | **AST** | **ALT** | **ALP** | **TBA** | **T-Bili** | **D- Bili** | **SOD** | **PA** |
| --- | --- | --- | --- | --- | --- | --- | --- | --- |
|  | **(U/L)** | **(U/L)** | **(U/L)** | **(µmol/L)** | **(µmol/L)** | **(µmol/L)** | **(U/mL)** | **(mg/L)** |
| Control | 99.75±8.73 | 33.75±2.95 | 150.29±23.03 | 11.86±2.94 | 19.19±0.20 | 5.71±0.96 | 91.00±7.21 | 8.13±1.17 |
| Model | 137.75±23.64^*^ | 41.25±4.60 | 190.00±36.88^*^ | 21.27±4.89^**^ | 21.25±0.51^*^ | 7.18±0.90^*^ | 80.50±5.62^*^ | 6.63±1.41^*^ |
| JGCC | 101.17±8.37^#^ | 34.60±4.22 | 173.00±33.03 | 16.05±2.88^#^ | 20.24±1.78 | 5.92±0.58^#^ | 83.33±8.18 | 8.71±1.91^#^ |
| ^*^P<0.05, ^**^P<0.01vs Control; ^#^P<0.05, ^##^P<0.01vs Model. | | | | | | | | |
