# Supplementary material for: Metabolomics Analysis Coupled With UPLC/MS on Therapeutic Effect of Jigucao Capsule Against Dampness-Heat Jaundice Syndrome
Source: Front Pharmacol. 2022 Jan 28;13:822193. doi: 10.3389/fphar.2022.822193 (PMC8831696; doi:10.3389/fphar.2022.822193)
Supplement: Supplementary file 2 [file Table2.docx]

**Table S2** Secondary fragmentation of potential biomarkers in the urine of dampness-heat jaundice model rats

| **NO** | **Rt** | **m/z determined** | **Proposed Composition** | **Postulated Identity** | **MS/MS** | **Structural Formula** |
| --- | --- | --- | --- | --- | --- | --- |
| 1 | 0.82 | 173.0084 | C_6_H_6_O_6_ | *Cis*-Aconitic acid | 173.0458[M-H]^-^, 129.0578[M-H-CO_2_]^-^, 111.0492[M-H-CH_2_O_3_]^-^, 85.0714[M-H-C_2_O_4_]^-^ | 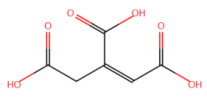 |
| 2 | 1.02 | 267.1098 | C_5_H_10_O_4_ | 2,3-Dihydroxyvaleric acid | 267.1389[2M-H]^-^, 187.1087[2M-H-CH_4_O_4_]^-^ | 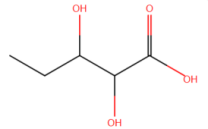 |
| 3 | 1.51 | 299.1665 | C_19_H_24_O_3_ | 2-Methoxyestrone | 299.1602[M-H]^-^, 251.1118[M-H-C_2_H_8_O]^-^ | 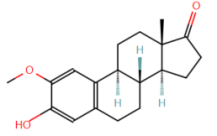 |
| 4 | 1.58 | 139.0509 | C_6_H_6_N_2_O_2_ | Urocanic acid | 139.0513[M+H]^+^,122.0280[M+H-NH_3_]^+^, 96.0481[M+H-C_2_H_5_N]^+^ | 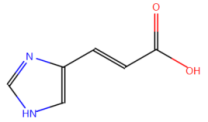 |
| 5 | 1.78 | 292.1054 | C_10_H_17_NO_6_ | Malonylcarnitine | 292.1316[M+FA-H]^-^, 188.0419[M+FA-H-C_3_H_4_O_4_]^-^, 156.1086[M+FA-H-C_5_H_14_O_3_N]^-^ | 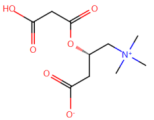 |
| 6 | 2.06 | 312.1089 | C_11_H_21_NO_7_S | N-(1-Deoxy-1-fructosyl)methionine | 312.1133[M+H]^+^,276.1470[M+H-2H_2_O]^+^, 229.1613[M+H-2H_2_O-CH_3_S]^+^ | 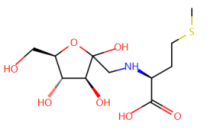 |
| 7 | 3.15 | 165.0559 | C_9_H_8_O_3_ | Phenylpyruvic acid | 165.0565[M+H]^+^, 137.0597[M+H-C_2_H_4_]+ | 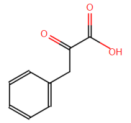 |
| 8 | 3.18 | 180.0883 | C_6_H_13_NO_5_ | Glucosamine | 180.0923[M+H]^+^,162.0623[M+H-H_2_O]^+^, 144.0488[M+H-2H_2_O]^+^ | 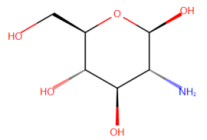 |
| 9 | 3.95 | 224.0560 | C_9_H_9_NO_3_ | Adrenochrome | 224.0872[M+FA-H]^-^, 193.0758[M+FA-H-CH_3_O]^-^ | 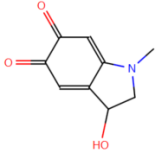 |
| 10 | 5.16 | 338.0888 | C_15_H_17_NO_8_ | 5-Hydroxy-6-methoxyindole glucuronide | 338.0869[M-H]^-^,175.0228[M-H-C_9_H_9_NO_2_]^-^, 162.0543[M-H-C_6_H_8_O_6_]^-^ | 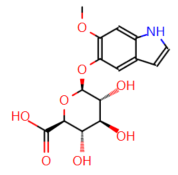 |
| 11 | 5.22 | 250.0717 | C_11_H_11_NO_3_ | 5-Methoxyindoleacetate | 250.0707[M+FA-H]^-^, 206.0789[M+FA-H-CO_2_]^-^ | 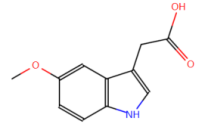 |
| 12 | 5.93 | 146.0607 | C_9_H_7_NO | 1H-Indole-3-carboxaldehyde | 146.0629[M+H]^+^, 119.0891[M+H-CHN]^+^ | 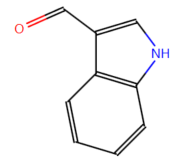 |
| 13 | 6.04 | 245.1749 | C_12_H_24_O_2_ | Dodecanoic acid | 245.1747[M+FA-H]^-^, 201.1508[M+FA-H-CO_2_]^-^, 173.1205[M+FA-H-C_3_H_4_O_2_]^-^ | 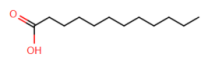 |
| 14 | 6.73 | 333.1129 | C_8_H_9_NO_3_ | Pyridoxal | 333.1052[2M-H]^-^,297.1392[2M-H-2H_2_O]^-^, 283.0883[2M-H-CH_6_O_2_]^-^ | 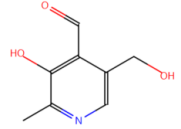 |
| 15 | 7.19 | 321.1579 | C_10_H_11_NO | Tryptophanol | 321.1779[2M-H]^-^,285.1422[2M-H-2H_2_O]^-^, 245.1678[2M-H-C_3_H_8_O_2_]^-^ | 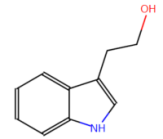 |
| 16 | 7.37 | 263.1119 | C_10_H_16_N_4_O_3_ | Homocarnosine | 263.1129[M+Na]^+^,245.1223[M+Na-H_2_O]^+^, 133.0670[M+Na-C_5_H_8_NO_3_]^+^ | 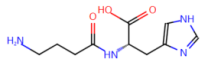 |
| 17 | 7.43 | 243.1600 | C_12_H_22_O_2_ | *Trans*-Dodec-2-enoic acid | 243.1411[M+FA-H]^-^, 113.0649[M+FA-H-C_7_H_14_O_2_]^-^ | 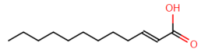 |
| 18 | 7.47 | 593.3384 | C_33_H_44_N_4_O_6_ | Mesobilirubinogen | 593.8547[M+H]^+^, 533.3176[M+H-C_2_H_4_O_2_]^+^ | 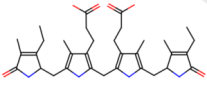 |
| 19 | 7.49 | 245.1383 | C_12_H_22_O_5_ | 3-Hydroxydodecanedioic acid | 245.1678[M-H]^-^,199.1365[M-H-CH_2_O_2_]^-^, 155.1497[M-H-C_2_H_2_O_4_]^-^ | 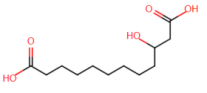 |
| 20 | 7.80 | 377.0771 | C_10_H_7_NO_3_ | Kynurenic acid | 377.0891[2M-H]^-^, 243.0661[2M-H-C_7_H_4_NO_2_]^-^, 233.1867[2M-H-C_9_H_6_NO]^-^ | 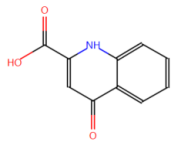 |
| 21 | 8.09 | 595.3488 | C_33_H_4_6N_4_O_6_ | *L*-Urobilin | 595.3667[M+H]^+^, 410.1938[M+H-C_9_H_15_O_3_N]^+^, | 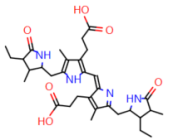 |
| 22 | 8.66 | 327.2318 | C_20_H_32_O_2_ | Arachidonic acid | 327.2346[M+Na]^+^,291.1256[M+Na-2H_2_O]^+^, 267.1656[M+Na-C_2_H_4_O_2_]^+^ | 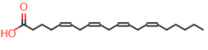 |
| 23 | 8.99 | 407.2786 | C_24_H_38_O_5_ | 7-Ketodeoxycholic acid | 407.2189[M+H]^+^,345.2566[M+H-C_2_H_6_O_2_]^+^, 315.2454[M+H-C_3_H_8_O_3_]^+^ | 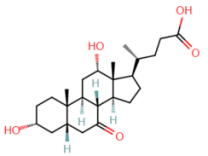 |
| 24 | 9.02 | 503.2353 | C_23_H_38_O_7_S | 3-Sulfodeoxycholic acid | 503.2355[M+FA-H]^-^,441.1827[M+FA-H-CH_2_O_3_]^-^, 401.1044[M+FA-H_6_O_4_S]^-^ | 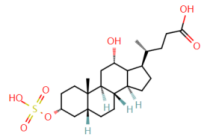 |
| 25 | 9.17 | 405.2635 | C_24_H_36_O_5_ | 7a,12a-Dihydroxy-3-oxo-4-cholenoic acid | 405.2651[M+H]^+^, 290.2769[M+H-C_6_H_11_O_2_]^+^ | 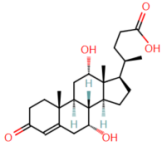 |
