# Supplementary material for: Metabolomics Analysis Coupled With UPLC/MS on Therapeutic Effect of Jigucao Capsule Against Dampness-Heat Jaundice Syndrome
Source: Front Pharmacol. 2022 Jan 28;13:822193. doi: 10.3389/fphar.2022.822193 (PMC8831696; doi:10.3389/fphar.2022.822193)
Supplement: Supplementary file 3 [file Table3.docx]

**Table S3** Identification of related marker metabolites of Jigucao capsule based on urine metabolic spectrum

| **NO.** | **Rt** | **Metabolite** | **Molecular** | **Ion** | **m/z** | **Error** | **T’TEST** | **Trend** | **JGCC** |
| --- | --- | --- | --- | --- | --- | --- | --- | --- | --- |
|  |  | **Name** | **Formula** | **form** | **determined** | （mDa） |  |  |  |
| 1 | 0.82 | *Cis*-Aconitic acid | C_6_H_6_O_6_ | [M-H]^-^ | 173.0084 | -0.2 | 0.007790 | ↑ | - |
| 2 | 1.02 | 2,3-Dihydroxyvaleric acid | C_5_H_10_O_4_ | [2M-H]^-^ | 267.1098 | 1.8 | 0.039910 | ↑ | √ |
| 3 | 1.51 | 2-Methoxyestrone | C_19_H_24_O_3_ | [M-H]^-^ | 299.1665 | -4.5 | 0.027107 | ↑ | √ |
| 4 | 1.58 | Urocanic acid | C_6_H_6_N_2_O_2_ | [M+H]^+^ | 139.0509 | 0.5 | 0.013914 | ↓ | √^##^ |
| 5 | 1.78 | Malonylcarnitine | C_10_H_17_NO_6_ | [M+FA-H]^-^ | 292.1054 | 2.2 | 0.037834 | ↓ | - |
| 6 | 2.06 | N-(1-Deoxy-1-fructosyl)methionine | C_11_H_21_NO_7_S | [M+H]^+^ | 312.1089 | 1.6 | 0.046442 | ↑ | √ |
| 7 | 3.15 | Phenylpyruvic acid | C_9_H_8_O_3_ | [M+H]^+^ | 165.0559 | 0.7 | 0.004537 | ↑ | - |
| 8 | 3.18 | Glucosamine | C_6_H_13_NO_5_ | [M+H]^+^ | 180.0883 | 1.1 | 0.040401 | ↑ | √^#^ |
| 9 | 3.95 | Adrenochrome | C_9_H_9_NO_3_ | [M+FA-H]^-^ | 224.0560 | 0.1 | 0.000361 | ↑ | √^##^ |
| 10 | 5.16 | 5-Hydroxy-6-methoxyindole glucuronide | C_15_H_17_NO_8_ | [M-H]^-^ | 338.0888 | 0.7 | 0.021314 | ↓ | - |
| 11 | 5.22 | 5-Methoxyindoleacetate | C_11_H_11_NO_3_ | [M+FA-H]^-^ | 250.0717 | 0.2 | 0.000002 | ↑ | √ |
| 12 | 5.93 | 1H-Indole-3-carboxaldehyde | C_9_H_7_NO | [M+H]^+^ | 146.0607 | 2.3 | 0.003512 | ↓ | √ |
| 13 | 6.04 | Dodecanoic acid | C_12_H_24_O_2_ | [M+FA-H]^-^ | 245.1749 | -0.6 | 0.023037 | ↑ | √^#^ |
| 14 | 6.73 | Pyridoxal | C_8_H_9_NO_3_ | [2M-H]^-^ | 333.1129 | -3.5 | 0.007254 | ↑ | √^#^ |
| 15 | 7.19 | Tryptophanol | C_10_H_11_NO | [2M-H]^-^ | 321.1579 | -2.4 | 0.001160 | ↑ | √^##^ |
| 16 | 7.37 | Homocarnosine | C_10_H_16_N_4_O_3_ | [M+Na]^+^ | 263.1119 | 0.9 | 0.009323 | ↑ | - |
| 17 | 7.43 | *Trans*-Dodec-2-enoic acid | C_12_H_22_O_2_ | [M+FA-H]^-^ | 243.1600 | 0.4 | 0.016868 | ↑ | √^#^ |
| 18 | 7.47 | Mesobilirubinogen | C_33_H_44_N_4_O_6_ | [M+H]^+^ | 593.3384 | 4.5 | 0.006352 | ↓ | √ |
| 19 | 7.49 | 3-Hydroxydodecanedioic acid | C_12_H_22_O_5_ | [M-H]^-^ | 245.1383 | -0.6 | 0.014050 | ↑ | √ |
| 20 | 7.80 | Kynurenic acid | C_10_H_7_NO_3_ | [2M-H]^-^ | 377.0771 | -0.3 | 0.037443 | ↑ | √ |
| 21 | 8.09 | *L*-Urobilin | C_33_H_46_N_4_O_6_ | [M+H]^+^ | 595.3488 | -0.8 | 0.024033 | ↑ | √^#^ |
| 22 | 8.66 | Arachidonic acid | C_20_H_32_O_2_ | [M+Na]^+^ | 327.2318 | 4.6 | 0.039426 | ↑ | √^#^ |
| 23 | 8.99 | 7-Ketodeoxycholic acid | C_24_H_38_O_5_ | [M+H]^+^ | 407.2786 | -1.1 | 0.014785 | ↑ | - |
| 24 | 9.02 | 3-Sulfodeoxycholic acid | C_23_H_38_O_7_S | [M+FA-H]^-^ | 503.2353 | 4.0 | 0.005663 | ↑ | √ |
| 25 | 9.17 | 7a,12a-Dihydroxy-3-oxo-4-cholenoic acid | C_24_H_36_O_5_ | [M+H]^+^ | 405.2635 | 1.0 | 0.044066 | ↑ | √ |
| ↓：Decreased,↑：Increased, Model vs Control. -: Compared with the model, JGCC group was no tendency to approach the control group; √: Compared with the model, JGCC group showed a tendency to approach the control group; (^#^P<0.05; ^##^P<0.01). | | | | | | | | | |
